# Feasibility and Preliminary Efficacy of Web-Based and Mobile Interventions for Common Mental Health Problems in Working Adults: Multi-Arm Randomized Pilot Trial

**DOI:** 10.2196/34032

**Published:** 2022-03-03

**Authors:** Marcos Economides, Heather Bolton, Rhian Male, Kate Cavanagh

**Affiliations:** 1 Unmind Ltd London United Kingdom; 2 School of Psychology University of Sussex East Sussex United Kingdom

**Keywords:** mHealth, workplace, CBT, ACT, feasibility, stress, anxiety, depression, resilience, mobile phone

## Abstract

**Background:**

There is growing interest in digital platforms as a means of implementing scalable, accessible, and cost-effective mental health interventions in the workplace. However, little is known about the efficacy of such interventions when delivered to employee groups.

**Objective:**

This study aims to evaluate the feasibility and preliminary efficacy of a digital mental health platform for the workplace, which incorporates evidence-based practices such as cognitive behavioral therapy and acceptance and commitment therapy. A total of 3 brief, unguided interventions designed to address stress, anxiety, and resilience, respectively, are evaluated. The primary aim is to determine the feasibility of the study methods and interventions in preparation for a definitive randomized controlled trial.

**Methods:**

The study used a fully remote, parallel, multi-arm, external pilot randomized controlled trial, with 3 intervention arms and a no-intervention control group. Participants were working adults representative of the general UK population with respect to age, sex, and ethnicity who were recruited from a web-based participant platform. Primary outcomes included objective and self-report measures of feasibility, acceptability, engagement, transferability, relevance, and negative effects. Secondary outcomes included 4 self-report measures of mental health and well-being, completed at baseline (time point 0 [t0]), postintervention (time point 1 [t1]), and the 1-month follow-up (time point 2 [t2]). Secondary outcomes were analyzed via linear mixed-effects models using intention-to-treat principles. Preregistered criteria for progression to a definitive trial were evaluated.

**Results:**

Data were collected between January and March of 2021. A total of 383 working adult participants meeting trial eligibility were randomized, of whom 356 (93%) were retained at t2. Objective engagement data showed that 67.8% (196/289) of participants randomized to an intervention arm completed their intervention. Overall, 87.1% (203/233) of participants reported being *satisfied* or *very satisfied* with their intervention and rated the quality of their intervention as *good* or *excellent*. All intervention groups reported significantly greater improvements than the control group on at least one secondary outcome at t1, with between-group Hedges *g* effect sizes for the pooled interventions ranging from 0.25 (95% CI 0.05-0.46) to 0.43 (95% CI 0.23-0.64). All the improvements were maintained at t2.

**Conclusions:**

The study methods were feasible, and all preregistered criteria for progression to a definitive trial were met. Several minor protocol amendments were noted. Preliminary efficacy findings suggest that the study interventions may result in improved mental health outcomes when offered to working adults.

**Trial Registration:**

ISRCTN Registry 80309011; http://www.isrctn.com/ISRCTN80309011

## Introduction

### Background and Rationale

Mental illness affects hundreds of millions of people worldwide, resulting in decreased quality of life, family and community disruption, increased health care costs, and a significant economic burden for employers [1,2]. Employee performance, rates of illness, absenteeism, and staff turnover are all affected by employees’ mental health status. In the United Kingdom, workplace mental health problems result in an estimated 70 million lost workdays and a total cost of up to £45 billion (US $61 billion) each year for businesses [3]. This is compounded by an estimated global treatment gap of >50% for people with mental health disorders [4,5].

There is growing interest in web and smartphone apps as a means of increasing the reach of mental health and well-being interventions [6,7]. Digital platforms can offer a broad range of content within a standardized environment that is interactive and dynamic while also being widely accessible, cost-efficient, and nonstigmatizing. With fewer access barriers, digital platforms also have the potential to offer a preventative solution to common mental health problems by facilitating sustained, proactive engagement [8,9]. Such platforms can vary widely in their means of delivery (web vs mobile app), the core therapeutic approach they use (with cognitive behavioral therapy [CBT], mindfulness meditation [MM], and positive psychology being common), and the duration and format of their content.

There is now convincing evidence for the effectiveness of digital interventions when delivered in health and community settings [10], as well as emerging evidence that they may be effective when delivered in occupational settings [11,12]. Previous meta-analyses have found small positive effects on psychological well-being (Hedges *g*=0.37) and work effectiveness (Hedges *g*=0.25) [11] and small to moderate effects on common mental health outcomes, such as stress (Hedges *g*=0.54), anxiety (Hedges *g*=0.34), and symptoms of depression (Hedges *g*=0.30) [12]. However, the current evidence base is limited by considerable heterogeneity across studies and an insufficient number of high-quality trials. Moreover, only a fraction of for-profit mental health apps (MHapps) are supported by empirical evidence [13], with added concerns that such platforms are frequently characterized by low adherence [14-16]. Together, these suggest the need for further research.

In this study, we conduct an external pilot randomized controlled trial (RCT) as part of the initial testing of *Unmind*—a novel digital mental health platform for the workplace. *Unmind* provides employees with tools to help them track, maintain, and improve their mental health and well-being. It features a broad range of content that draws on multiple evidence-based approaches such as CBT [17], MM [18], behavioral activation [19], acceptance and commitment therapy (ACT [20]), and positive psychology [21]. Central to the platform are individual learning and development courses (known as *Series*) designed to address specific topics of mental health and well-being. *Series* are short, standalone interventions, typically ranging between 5 and 7 sessions, each of approximately 10 minutes in duration, and can feature a mix of audio and video content, infographics, and interactions with a chatbot.

### Study Objective

Consistent with recent guidelines on pilot trials [22,23], the primary aim of this study is to evaluate the feasibility of the study methods, and 3 separate *Unmind Series* that address the topics of stress, anxiety, and resilience, respectively, in preparation for a future definitive RCT. We chose to evaluate content relating to stress and anxiety as these are highly prevalent in the workplace [3] and have been extensively studied in previous evaluations of MHapps [24-26], allowing for a comparison of the current findings to previous evidence. In addition, we chose to evaluate content relating to resilience, as evidence suggests that it plays an important role in the prevention of mental health problems [27] and thus may be integral to the effectiveness of a preventative platform. A secondary aim is to establish the preliminary efficacy of each intervention with respect to self-report measures of stress, anxiety, symptoms of depression, and resilience, including establishing between-group effect sizes and 95% CIs (for each intervention compared with the control group). Although depression was not a specific target of any of the study interventions, we chose to include it as an outcome as it is highly comorbid with stress and anxiety [28,29] and a common problem in workplace settings [3]. Finally, we also aim to report on the combined effects of all interventions compared with the control group.

As the *Unmind* app comprises an extensive library of standalone interventions, it is important that each component of the app is evaluated. We chose to include 3 intervention arms in this study as this is more efficient than performing sequential 2-arm trials and increases the proportion of participants randomized to an intervention arm [30]. In addition, if a definitive RCT is warranted, an aim might be to evaluate whether each intervention arm has a greater effect on the specific outcome targeted by that intervention relative to the other intervention arms. Thus, this study uses a parallel, multi-arm, external pilot RCT design and recruited UK-based, community-dwelling, working adult participants who are randomly allocated to 1 of 3 intervention arms or to a no-intervention control group. We chose to implement a no-intervention control as (1) participants were not selected on the basis of poor mental health or seeking help for a problem, (2) recent evidence suggests that wait-list groups may introduce nocebo effects in psychotherapy trials [31], and (3) participants received monetary compensation for taking part.

The feasibility of each intervention arm is assessed via objective and self-reported outcomes capturing recruitment, retention, intervention uptake and adherence, acceptability, transferability, relevance, and negative effects. The preliminary efficacy of each intervention arm is assessed via self-report outcome measures delivered before (time point 0 [t0]) and after the interventions (time point 1 [t1]; 2 weeks after t0) and at the 1-month follow-up (time point 2 [t2]). The results of this study are intended to inform whether a definitive RCT to evaluate the efficacy of each intervention arm is warranted and provide estimates of the parameters required for its design and implementation.

## Methods

The authors followed the CONSORT (Consolidated Standards of Reporting Trials) 2010 guidelines [32] when preparing this study, including recent extensions to pilot trials [22] and multi-arm trials [33].

### Trial Design

This study was a parallel, multi-arm, external pilot RCT with pre- (t0) and postintervention (t1; 2 weeks after t0) assessments and a 1-month follow-up (t2). Participants were randomly allocated to 1 of 3 brief, self-guided psychological interventions (*Series*) featured on the *Unmind* platform or to a no-intervention control group in a 1:1:1:1 allocation ratio. Participants were working adults recruited from the Prolific web-based recruitment platform [34], and the entire study was conducted on the web between January and March 2021. Of note, the study commenced several weeks after the start of a third national UK lockdown (in response to the SARS-CoV-2 pandemic), and t2 data were collected after the commencement of a phased easing of lockdown restrictions. The trial was preregistered at ISRCTN 80309011, and a full study protocol was preregistered at Open Science Framework in December 2020.

### Ethics Approval

The trial received ethical approval from the University of Sussex sciences and technology research ethics committee (ER/KC226/2).

### Participants

Participants were recruited via the Prolific web-based recruitment platform, which has been empirically tested across attributes such as participant response rates and data quality [35]. Inclusion criteria were (1) aged at least 18 years, (2) currently residing in the United Kingdom, (3) self-identifying as being in full- or part-time employment, (4) having an active account on Prolific, (5) having access to an internet connection via a smartphone or desktop device, and (6) being fluent in English. Prolific indicated that there were 50,978 eligible individuals at the time of conducting the study.

Prolific implements a prescreening system that allows researchers to screen for eligibility without implementing a screening questionnaire. Prolific also supports the recruitment of study samples representative of the national UK population with respect to age, sex, and ethnicity based on guidelines from the UK Office of National Statistics. This study drew upon this feature to maximize the generalizability of the findings.

### Procedures

All study assessments were hosted on the Qualtrics survey platform [36], and participants were required to provide informed consent via a form built into each study assessment alongside a digital information sheet. All participants who completed the t0 assessment were invited to complete the t1 and t2 assessments via the Prolific recruitment platform. Participants were offered a £7 (US $9.50) incentive for completing each of the 3 study assessments (baseline, postintervention, and 1-month follow-up), delivered via Prolific. Participants randomized to one of the intervention arms received reminder messages on days 5 and 10 of the intervention (delivered via Prolific’s anonymous inbox system), encouraging them to complete all intervention sessions. However, participant reimbursement was not contingent on intervention adherence.

### Randomization

Randomization occurred at the end of t0 and was implemented via the Qualtrics *randomizer* feature, which uses block randomization to ensure balanced groups. It was not possible to blind the participants to group assignments. After randomization, participants assigned to one of the intervention arms were sent a message via Prolific’s anonymous inbox system with instructions on how to access their intervention, including using a unique voucher code to sign up to the *Unmind* platform. The research team remained blind to group assignment for the duration of data collection but was unblinded during data analysis.

### Interventions

#### Overview

*Unmind* is a digital platform designed to be used by working adults to measure, manage, and improve their mental health and well-being. It can be accessed via the web, mobile, or tablet (Android or iOS), and the *Unmind* smartphone app can be downloaded via the Apple or Google Play stores. The platform features a wide range of resources and content created by academics and clinicians with expertise in adult mental health, which are rooted in evidence-based practices such as CBT [17], MM [18], behavioral activation [19], ACT [20], and positive psychology [21].

**Figure 1:**
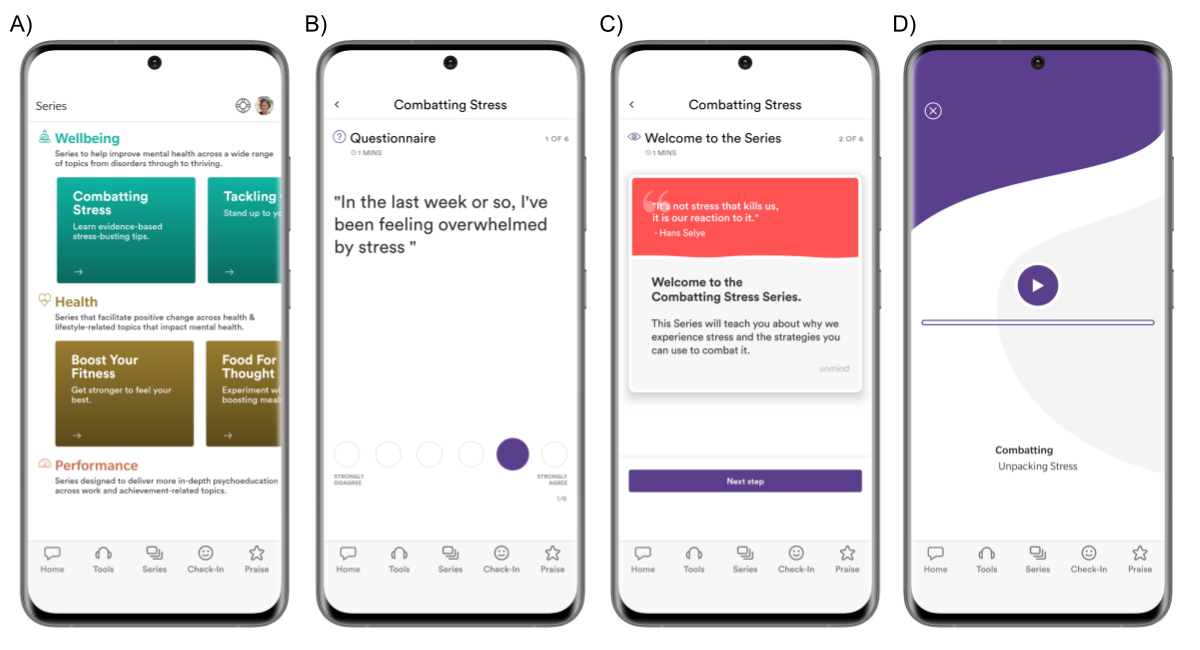
Screenshots of the *Unmind* smartphone app showing the *Series* tab (panel A) and examples from the *combatting stress* intervention (panels B-D).

Although *Unmind* includes a wide range of content and features, this study focused on evaluating *Series*. *Series* are brief, unguided learning and development courses, typically comprising between 5 and 7 sessions, each of approximately 10 minutes in duration, that are designed to be completed sequentially, and include a mix of audio and video content, infographics, and interaction with a chatbot (see Figure 1 for example screenshots). Each *Series* focuses on a specific symptom, topic, trait, or behavior related to mental health and typically uses a key therapeutic approach, such as CBT, MM, or ACT. *Series* are designed to provide both reactive support (to manage or address an existing problem) and proactive support (to prevent the onset of a future mental health problem).

The intervention arms in this study comprised 3 individual *Series* designed to address stress, anxiety, and resilience, respectively. For the purposes of the study, participants were instructed to only engage with their allocated intervention, despite having access to the full *Unmind* platform, and were excluded from standard email campaigns that encourage interaction with content not evaluated in this study. Participants had 2 weeks to complete their allocated intervention and were free to progress through the intervention at their own pace. A description of each intervention arm is provided in the following sections.

#### Combatting Stress

This intervention draws upon CBT and ACT techniques and is designed to help users better manage their day-to-day stressors. Over the course of 7 sessions, it provides psychoeducation on stress and its physical manifestations, helps users spot personal triggers, and explores different approaches to coping. It also introduces the idea of acceptance. Users are taught stress management techniques and are encouraged to practice between sessions.

#### Working With Worry

This intervention is underpinned by theoretical models of generalized anxiety disorder (GAD), although it is targeted at users who identify as worriers rather than those meeting any predefined criteria for a diagnosis of GAD. Content spans 7 sessions and covers key elements of CBT, including tolerance of uncertainty, challenging worry beliefs, problem solving, and working with imagery. It also encourages users to apply evidence-based techniques, including relaxation and attentional focus.

#### Building Resilience

This intervention aims to help users apply evidence-based techniques to aid the cultivation of essential qualities of personal resilience, drawing upon CBT and ACT. Over the course of 7 sessions, learning covers topics such as honing strengths, facing challenges, and tolerating discomfort. It also explores aspects such as coping styles and realistic optimism. The *Series* encourages users to increase their self-awareness and guides them to build a personal resilience plan.

In each *Series*, learning is optimized by the use of a chatbot to allow note-taking and aid reflection, as well as the use of short recap videos at the beginning of each new session. Each *Series* also has its own accompanying brief handbook, which is emailed to participants as a PDF file on starting their first session. Handbooks contain a summary of key learning points and infographics from the *Series* and include space for participants to write any further reflections on their learning from each session.

For the purposes of this study, participants were instructed not to engage with other content and features included in the *Unmind* app (and not described here) so that the feasibility of the study interventions could be assessed in isolation. The app also includes a *Help* page containing information and resources for key well-being topics and signposting to urgent problems. Participants had access to versions 2.56.0 to 2.59.0 of the *Unmind* app, and no major app changes or updates were launched during the 2-week study period.

### Outcomes

#### Overview

Participant demographics and other variables were captured at t0, including whether each participant had engaged with therapy or counseling, and a mental health or well-being app within 6 months before taking part in the study. Participants were also asked to rate the extent to which they agreed with the statement, “Do you agree that it’s important for people to look after their mental health and wellbeing?” on a 5-point Likert scale from *strongly agree* to *strongly disagree*.

#### Primary Outcome Measures

Recent guidelines suggest that complex health interventions should be feasible, acceptable, engaging, transferable to other settings, and relevant [37]. In addition, psychological interventions should be evaluated for negative effects [38]. Therefore, preregistered primary outcomes included the following:

1. Feasibility: recruitment, intervention uptake, and retention (at t1 and t2)
2. Acceptability: intervention adherence and completion rates, participant satisfaction, and reasons for discontinuing the intervention
3. Engagement: average sessions completed and 3 questions adapted from the Mobile App Rating Scale [39]
4. Transferability: 1 question adapted from the Mobile App Rating Scale
5. Relevance: 1 question assessing subjective relevance
6. Negative effects: 1 question adapted from recent guidelines on assessing negative effects [40] and the proportion of participants that reliably deteriorated across all secondary outcome measures.

Outcomes were measured through a combination of objective data (captured by the *Unmind* platform) and self-reported data captured at t1 (Multimedia Appendix 1).

#### Secondary Outcome Measures

Preregistered secondary outcomes included self-report measures capturing symptoms of common mental health problems.

##### The Perceived Stress Scale-10

The Perceived Stress Scale (PSS) is a 10-item scale that asks respondents to rate how often they feel or think that their lives are unpredictable, uncontrollable, and overloaded on a 5-point Likert scale from 0 (never) to 4 (very often) [41]. Total scores range from 0 to 40, with higher scores indicating greater perceived stress. The PSS has a Cronbach α >.70 across 12 individual studies (and .91 in this study at t0) and good test–retest reliability across 4 individual studies [42]. The original scale uses a 1-month reporting period; however, this has been shortened in several previous studies [43,44], and this study used a 2-week reporting period.

##### The GAD-7 Scale

The GAD-7 is a 7-item scale used to screen for the presence and severity of an anxiety disorder [45]. Participants rate each item on a 4-point Likert scale from 0 (not at all) to 3 (nearly every day), with total scores ranging from 0 to 21. A score ≥10 is suggestive of the presence of anxiety, and scores of 5, 10, and 15 are taken as cutoff points for mild, moderate, and severe anxiety, respectively. The GAD-7 has excellent reliability and internal consistency (Cronbach α of .89 in the original validation and .91 in this study at t0) and has been validated in both the general population and primary care settings [45,46].

##### The Patient Health Questionnaire-8

The Patient Health Questionnaire (PHQ)-8 is an 8-item scale derived from the PHQ-9, which screens for the presence and severity of depression [47]. The PHQ-8 omits an item that assesses suicidal ideation and is more appropriate for use in nonclinical samples and settings [48]. The response options are equivalent to those of the GAD-7, with total scores ranging from 0 to 24. A score ≥10 suggests the presence of depression, and scores of 5, 10, 15, and 20 are taken as cutoff points for mild, moderate, moderately severe, and severe depression, respectively. The PHQ-8 has excellent internal consistency (Cronbach α of .89 in primary care settings and .88 in this study at t0) and excellent test–retest reliability [49].

##### Brief Resilience Scale

The Brief Resilience Scale (BRS) is a short, 6-item scale designed to assess people’s ability to bounce back or recover after stressful events [50]. Participants rate each item on a 5-point Likert scale from 1 (strongly disagree) to 5 (strongly agree) or the reverse for negatively worded items. The BRS is scored by reverse coding items 2, 4, and 6 and computing the mean of the 6 items. The creators of the scale have suggested that scores <3 be interpreted as *low* resilience and scores ≥4.3 be interpreted as *high* resilience [51]. The BRS displays good internal consistency (Cronbach α=.80-.91 and .91 in this study at t0) and test–retest reliability.

### Progression Criteria

As per formal guidelines [52], preregistered progression criteria were defined as follows: (1) full study recruitment within 1 month; (2) at least 30% intervention completion rates based on a previous meta-analysis of adherence to unguided psychological interventions [53]; (3) at least 75% adherence to protocol instructions (defined as the proportion of participants who refrain from engaging with ≥1 *Series* session outside of their allocated *Series*); (4) at least 50% of participants reporting being *satisfied* or *very satisfied* with the intervention and rating the quality of the intervention as *good* or *excellent*; and (5) the 95% CI on between-group effect sizes for secondary outcomes including at least a small effect (Hedges *g*=0.2) for ≥1 outcome measures.

Progression criteria were considered for each intervention arm individually.

### Sample Size

This study was powered for CIs on the feasibility outcomes. A sample size calculation indicated that approximately 100 participants were required to estimate feasibility outcomes with a margin of error ≤10% (based on a conservative population proportion of 50% for retention and adherence, and a 95% CI). This is consistent with previous guidelines suggesting that 60 to 100 participants per intervention arm are optimal for estimating binary outcomes in pilot RCTs [54]. Therefore, we aimed to recruit 400 participants in total.

### Statistical Methods

The results from all preregistered primary and secondary measures are reported. Minor deviations from the preregistered data analysis plan are reported in Multimedia Appendix 2 [40,41,55].

#### Primary Analyses

Descriptive statistics were used to report primary outcomes. Categorical data were reported as proportions in each response category, and Fisher exact test of independence was used to compare responses between intervention arms (with *P* values computed using Monte Carlo simulation and 2000 iterations). Where tests were significant, post hoc pairwise comparisons between study arms were performed (using false discovery rate methods to adjust *P* values).

Objective in-app usage data were provided by *Unmind*. For simplicity, intervention sessions were only characterized as *complete* if all components of the session were played. Descriptive statistics were used to characterize engagement and stratify participants according to whether they completed, started but did not complete, or failed to start their allocated intervention.

We computed the proportion of participants who self-reported reliable deterioration in mental health scores from t0 to t1, and t1 to t2, based on an estimate of the reliable change index for each outcome measure. The reliable change index was computed based on methods provided by Jacobson and Truax [56], using Cronbach α as a measure of reliability and an α level of .05 (Multimedia Appendix 3). Participants with missing data or those who were unable to reliably deteriorate based on t0 scores were excluded from this analysis.

#### Secondary Analyses

Secondary outcome measures were analyzed using both intention-to-treat (ITT) and per-protocol (PP) approaches. For the ITT analysis, all participants with complete t0 data were included, regardless of intervention adherence and any deviation from instructions. Participants were excluded from the PP analysis if they failed to complete all 7 intervention sessions, if they started an *Unmind Series* outside of their allocated intervention, or if they were lost to follow-up at t1. As findings from the PP analysis were largely equivalent to ITT, we opted to omit these results (although a comparison of effect sizes is reported in Multimedia Appendix 4).

Analyses were performed using linear mixed-effects models (LMMs) with restricted information maximum likelihood estimation (via the *lme4* package in R [57]). Each model included a within-subject factor *time* (with levels: t0, t1, and t2), a between-subject factor *group* (*combatting stress*, *working with worry*, *building resilience*, or control), their interaction as fixed effects, and a separate baseline for each participant. Time was modeled as a categorical factor. Model residuals were checked via *Q*–*Q* plots to assess model assumptions and goodness of fit. For each outcome, we reported (1) the estimated marginal means (EMMs) with 95% CIs for each time point and intervention arm, (2) *P* values for within-group contrasts comparing changes from t0 to t1 and t0 to t2, and (3) between-group contrasts (with 95% CIs) comparing changes from t0 to t1 and t0 to t2 for each intervention arm (and all intervention arms combined) relative to the control group (with both unadjusted and Tukey-adjusted *P* values). *P* values <.05 were considered significant. We also report a standardized effect size (Hedges *g* with 95% CI) for each between-group contrast. Hedges *g* was calculated using EMMs (as opposed to raw data, which require the use of complete cases only) and pooled SDs. The 95% CIs were calculated using equations 15 and 16 from Nakagawa and Cuthill [58]. *P* values were reported to a maximum of 3 decimal places, with values <.001 reported as *P*<.001.

#### Subgroup Analyses

Subgroup analyses were performed to examine changes in secondary outcome measures for participants who self-reported having at least mild symptoms at t0 or at least moderately low resilience. Thresholds for subgroup analyses were as follows: a score ≥16 on the PSS, ≥5 on the GAD-7, ≥5 on the PHQ-8, and <3 on the BRS. For simplicity, we report a comparison of Hedges *g* effect sizes for these subgroups versus the ITT analysis but omit the full output of each LMM. In addition, we analyzed the intervention feedback ratings for these subgroups separately. As the findings were similar to the ITT sample, these are reported in Multimedia Appendix 5.

Finally, multivariate logistic regression was conducted on the intervention group data only to explore whether any baseline variables were predictive of intervention completion (defined as *1* for randomized participants who completed all sessions of their allocated intervention and *0* for all other randomized participants). Predictor variables included all demographic variables and other baseline characteristics, as well as all self-report secondary outcome measures at t0. For categorical predictors, categories that included <10 observations were dropped from the regression analysis.

## Results

### Participants

Participant demographics and other baseline variables are presented in Table 1. The mean age of the participants was 44.6 (SD 14.3) years, 52% (199/383) were female, and 81.2% (311/383) were White, suggesting that the study sample was broadly representative of the general UK population [59]. All participants were employed (part-time: 94/383, 24.5%; self-employed: 49/383, 12.8%) across a broad range of industries and most had not used an MHapp (306/383, 79.9%) or not engaged in talking therapy (352/383, 91.9%) in a 6-month period before taking part in the study. Almost all participants (372/383, 97.1%) either *agreed* or *strongly agreed* with the statement, “It’s important for people to look after their mental health and wellbeing.”

**Table 1:** Participant demographics and baseline variables (N=383).

Variable		Overall	Study arm			
			Control (n=94)	CS^a^ (n=94)	WW^b^ (n=97)	BR^c^ (n=98)
Age (years), mean (SD; range)		44.6 (14.3; 18-75)	45.6 (14.2; 18-69)	44.8 (14.3; 19-75)	43.6 (14.7; 19-72)	44.7 (14.3; 18-69)
**Sex, n (%)**						
	Female	199 (52)	53 (56.4)	41 (43.6)	55 (56.7)	50 (51)
	Male	184 (48)	41 (43.6)	53 (56.4)	42 (43.3)	48 (49)
**Ethnicity, n (%)**						
	White^d^	311 (81.2)	76 (80.9)	79 (84)	74 (76.3)	82 (83.7)
	Black^e^	19 (5)	4 (4.3)	2 (2.1)	7 (7.2)	6 (6.1)
	Mixed or multiple	12 (3.1)	2 (2.1)	3 (3.2)	5 (5.2)	2 (2)
	Asian^f^	35 (9.1)	11 (11.7)	9 (9.6)	9 (9.3)	6 (6.1)
	Other^g^	6 (1.6)	1 (1.1)	1 (1.1)	2 (2.1)	2 (2)
**Employment, n (%)**						
	Full-time	238 (62.1)	60 (63.8)	63 (67)	55 (56.7)	60 (61.2)
	Part-time	94 (24.5)	26 (27.7)	18 (19.1)	28 (28.9)	22 (22.4)
	Self-employed	49 (12.8)	8 (8.5)	13 (13.8)	13 (13.4)	15 (15.3)
	Other	2 (0.5)	0 (0)	0 (0)	1 (1)	1 (1)
**Industry, n (%)**						
	Agriculture, forestry, or mining	1 (0.3)	1 (1.1)	0 (0)	0 (0)	0 (0)
	Industrials	22 (5.7)	4 (4.3)	9 (9.6)	3 (3.1)	6 (6.1)
	Energy or utilities	7 (1.8)	2 (2.1)	1 (1.1)	2 (2.1)	2 (2)
	Transport or logistics	17 (4.4)	2 (2.1)	5 (5.3)	6 (6.2)	4 (4.1)
	Media or creative industries	24 (6.3)	6 (6.4)	9 (9.6)	4 (4.1)	5 (5.1)
	Data or telecommunication	18 (4.7)	5 (5.3)	5 (5.3)	2 (2.1)	6 (6.1)
	Health care	40 (10.4)	13 (13.8)	6 (6.4)	12 (12.4)	9 (9.2)
	Education	63 (16.4)	17 (18.1)	17 (18.1)	12 (12.4)	17 (17.3)
	Life sciences	4 (1)	0 (0)	1 (1.1)	2 (2.1)	1 (1)
	Retail	32 (8.4)	2 (2.1)	11 (11.7)	8 (8.2)	11 (11.2)
	Hospitality, leisure, or travel	22 (5.7)	8 (8.5)	7 (7.4)	3 (3.1)	4 (4.1)
	Public or social service	30 (7.8)	8 (8.5)	6 (6.4)	7 (7.2)	9 (9.2)
	Finances, insurance, or real estate	22 (5.7)	5 (5.3)	4 (4.3)	8 (8.2)	5 (5.1)
	Professional services	25 (6.5)	7 (7.4)	4 (4.3)	8 (8.2)	6 (6.1)
	Other	56 (14.6)	14 (14.9)	9 (9.6)	20 (20.6)	13 (13.3)
**Education, n (%)**						
	None	4 (1)	0 (0)	2 (2.1)	1 (1)	1 (1)
	High school	138 (36)	30 (31.9)	33 (35.1)	36 (37.1)	39 (39.8)
	Undergraduate degree	172 (44.9)	46 (48.9)	41 (43.6)	42 (43.3)	43 (43.9)
	Postgraduate degree	69 (18)	18 (19.1)	18 (19.1)	18 (18.6)	15 (15.3)
**MHapp^h^ use (6 months), n (%)**						
	Yes	74 (19.3)	15 (16.0)	17 (18.1)	23 (23.7)	19 (19.4)
	No	306 (79.9)	78 (83.0)	76 (80.9)	73 (75.3)	79 (80.6)
	Maybe	3 (0.8)	1 (1.1)	1 (1.1)	1 (1)	0 (0)
**Therapy (6 months), n (%)**						
	Yes	31 (8.1)	6 (6.4)	3 (3.2)	12 (12.4)	10 (10.2)
	No	352 (91.9)	88 (93.6)	91 (96.8)	85 (87.6)	88 (89.8)
**Proactive** **MH^i^ care important, n (%)**						
	Strongly disagree	6 (1.6)	0 (0)	2 (2.1)	1 (1)	3 (3.1)
	Disagree	1 (0.3)	1 (1.1)	0 (0)	0 (0)	0 (0)
	Neither	4 (1)	0 (0)	1 (1.1)	1 (1)	2 (2)
	Agree	75 (19.6)	19 (20.2)	25 (26.6)	15 (15.5)	16 (16.3)
	Strongly agree	297 (77.5)	74 (78.7)	66 (70.2)	80 (82.5)	77 (78.6)

^a^CS: combatting stress.

^b^WW: working with worry.

^c^BR: building resilience.

^d^White British and other British.

^e^African, Caribbean, and Black British.

^f^Chinese, Indian, Bangladeshi, Pakistani, and other Asian.

^g^Arabian or any other ethnicity.

^h^MHapp: mental health app.

^i^MH: mental health.

Patient-reported outcome scores suggested that participants were, on average, experiencing mild symptoms of depression and anxiety at t0 (mean PHQ-8 6.9, SD 5.2; mean GAD-7 6.5, SD 5.1). The proportion of participants scoring above the cutoff for mild symptoms was 59.3% (227/383) for anxiety (GAD-7≥5) and 59.8% (229/383) for depression (PHQ-8≥5), whereas the proportion scoring above the cutoff for moderate symptoms was 26.9% (101/383) for anxiety (GAD-7≥10) and 28.2% (108/383) for depression (PHQ-8≥10). Self-reported stress levels were approximately consistent with population norms (mean PSS 17.0, SD 7.7 [55]).

### Primary Outcomes

#### Enrollment and Retention

**Figure 2:**
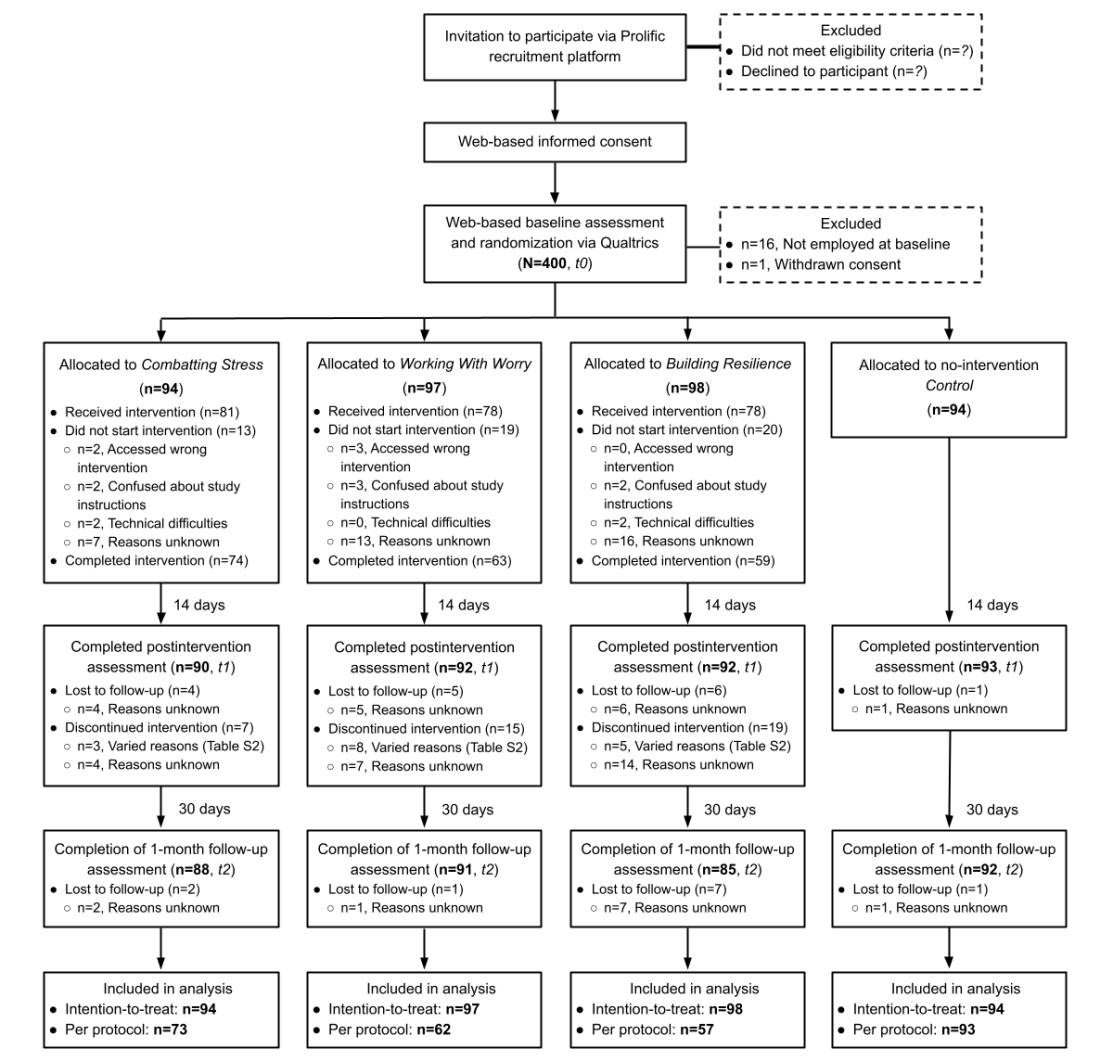
CONSORT (Consolidated Standards of Reporting Trials) flow of participants through the study. t0: time point 0; t1: time point 1; t2: time point 2.

The study was enrolled in January 2021 within 48 hours of launching the study advert. Figure 2 shows the participant flow through the trial. One of the participants withdrew consent after randomization, and 4% (16/400) of participants reported not being employed at t0 (in contrast to their prescreening responses) and were excluded from all analyses. Of the remaining 383 eligible participants, 367 (95.8%) completed an assessment at t1, and 356 (93%) completed an assessment at t2. Retention rates at t2 significantly differed across the intervention arms (*P*=.02; control 97.9%, *combatting stress* 93.6%, *working with worry* 93.8%, and *building resilience* 86.7%). Pairwise post hoc comparisons suggested significantly lower retention for *building resilience* than the control arm (adjusted *P*=.03); however, no other comparisons were significant. All groups exceeded the prespecified minimum retention for progression to a definitive trial.

#### Engagement and Adherence

##### Overview

A summary of the intervention engagement is shown in Table 2. Of the 289 participants randomized to an intervention, 237 (82%) started their allocated intervention, and 196 (67.8%) completed all intervention sessions. Of those who completed at least one session, 82.7% (196/289) proceeded to complete all sessions, which differed across intervention arms (*P*=.02). Pairwise post hoc comparisons suggested significantly higher completion for *combatting stress* (74/81, 91.4%) than for *building resilience* (59/78, 75.6%; adjusted *P*=.03) but not *working with worry* (63/78, 80.8%). Of the 289 participants randomized to an intervention, 47 (16.3%) did not start their intervention, and 5 (1.7%) incorrectly engaged with an intervention outside of the one they were allocated. Participants took an average of 6.38 (SD 4.10, range 0-15) days to complete all 7 intervention sessions, which did not significantly differ across intervention arms (*F*_2,193_=1.58; *P*=.21). Those who started but did not complete their allocated intervention completed a median of 3 out of 7 sessions (mean 3.24, SD 1.96, range 1-6). A summary of participants’ self-reported reasons for not starting or discontinuing their intervention can be found in Multimedia Appendix 6. The most common reasons for lack of engagement included insufficient time or technical difficulties (although these data were missing for participants who self-reported completing their intervention, regardless of objective adherence).

Overall, participants who objectively completed at least one intervention session self-reported spending a mean of 60.1 (median 60, SD 29.1, minimum 10, maximum 210) minutes on the *Unmind* platform, which differed significantly across groups (*F*_2,231_=3.12; *P*=.046). Post hoc Tukey tests suggested that participants in the *combatting stress* arm reported spending more time on the platform than participants in the *building resilience* arm (mean difference 11.49, 95% CI 0.64-22.34; adjusted *P*=.04) but not the *working with worry* arm (adjusted *P*=.40). Of the 274 participants who completed an assessment at t1, 177 (64.6%) reported receiving a handbook via email (*combatting stress* 58/90, 64%, *working with worry* 61/92, 66%, and *building resilience* 58/92, 63%) while completing their intervention. Of these 177 participants, 45 (25.4%) reported reading the entire handbook (*combatting stress* 12/58, 21%, *working with worry* 16/61, 26%, and *building resilience* 17/58, 29%), and 84 (47.5%) reported reading some of the handbook (*combatting stress* 27/58, 47%; *working with worry* 31/61, 51%; and *building resilience* 26/58, 45%). Overall, 4.2% (12/289) of participants deviated from the study instructions by engaging with ≥1 session outside their allocated intervention arm.

**Table 2:** Intervention adherence and engagement across the 3 intervention arms (N=289).

Variable		Overall	Study arm		
			CS^a^ (n=94)	WW^b^ (n=97)	BR^c^ (n=98)
**Completers**					
	Value, n	196	74	63	59
	Percentage of those randomized (95% CI)	67.8 (62.1-73.2)	78.7 (69.1-86.5)	64.9 (54.6-74.4)	60.2 (49.8-70)
	Percentage of those starting intervention (95% CI)	82.7 (77.3-87.3)	91.4 (83.0-96.5)	80.8 (70.3-88.8)	75.6 (64.6-84.7)
Days taken to complete intervention, mean (SD; range)		6.38 (4.10; 0-15)	6.59 (3.94; 0-15)	6.86 (3.85; 0-14)	5.61 (4.51; 0-14)
**Partial completers**					
	Value, n	41	7	15	19
	Percentage of those randomized (95% CI)	14.2 (10.4-18.8)	7.45 (3.1-14.7)	15.5 (8.9-24.2)	19.4 (12.1-28.6)
**Number of sessions completed by partial completers**					
	Values, mean (SD)	3.24 (1.96)	2.71 (1.80)	2.80 (1.47)	3.79 (2.27)
	Values, median (range)	3 (1-6)	3 (1-5)	3 (1-5)	4 (1-6)
**Did not start intervention**					
	Value, n	47	11	16	20
	Percentage of those randomized (95% CI)	16.3 (12.2-21)	11.7 (6-20)	16.5 (9.7-25.4)	20.4 (12.9-29.7)
**Engaged only with nonassigned intervention**					
	Value, n	5	2	3	0
	Percentage of those randomized (95% CI)	1.7 (0.6-4)	2.1 (0.3-7.5)	3.1 (0.6-8.8)	0 (0-3.7)
**Engaged with assigned and nonassigned interventions**					
	Value, n	12	4	5	3
	Percentage of those randomized (95% CI)	4.2 (2.2-7.1)	4.3 (1.2-10.5)	5.2 (1.7-11.6)	3.1 (0.6-8.7)

^a^CS: combatting stress.

^b^WW: working with worry.

^c^BR: building resilience.

##### Satisfaction and Feedback Ratings

Table 3 shows a summary of feedback ratings from participants who were retained at t1 and who also completed at least one intervention session based on objective app use (233/383, 60.8%; see Multimedia Appendix 1 for feedback questions). Importantly, most (203/233, 87.1%) participants were either *satisfied* (99/233, 42.5%) or *very satisfied* (104/233, 44.6%) with their intervention and rated the quality of their intervention as either *good* (96/233, 41.2%) or *excellent* (107/233, 45.9%). Feedback did not significantly differ across the intervention arms, except for the intervention quality (*P*=.02). Post hoc pairwise comparisons suggested that ratings differed between *working with worry* and *combatting stress* (adjusted *P*=.02) and differed marginally between *combatting stress* and *building resilience* (adjusted *P*=.07). Compared with *building resilience*, participants in the *combatting stress* arm were more likely to rate the intervention as *good* than *okay* or *poor*, and participants in the working with *worry* arm reported the highest ratio of *excellent* to *good ratings*.

Feedback ratings from participants scoring above predefined cutoffs for inclusion in subgroup analyses were largely equivalent to the overall sample and are included in Multimedia Appendix 5.

**Table 3:** Postintervention feedback ratings from participants who were retained at time point 1 (t1), both overall and for each intervention arm (N=233).

Feedback ratings		Overall n (%)	Study arm n (%)				*P* value^a^	
			CS^b^ (n=79)	WW^c^ (n=76)	BR^d^ (n=78)			
**Design of intervention**								.16
	Dull, not fun	1 (0.4)	0 (0)	0 (0)	1 (1.3)			
	Mostly boring	14 (6)	1 (1.3)	7 (9.2)	6 (7.7)			
	OK, fun enough	55 (23.6)	17 (21.5)	15 (19.7)	23 (29.5)			
	Moderately interesting and fun	103 (44.2)	41 (51.9)	32 (42.1)	30 (38.5)			
	Highly interesting and fun	60 (25.8)	20 (25.3)	22 (28.9)	18 (23.1)			
**Content of intervention**								.46
	Dull, not fun	3 (1.3)	0 (0)	1 (1.3)	2 (2.6)			
	Mostly boring	11 (4.7)	2 (2.5)	5 (6.6)	4 (5.1)			
	OK, fun enough	46 (19.7)	13 (16.5)	13 (17.1)	20 (25.6)			
	Moderately interesting and fun	94 (40.3)	35 (44.3)	28 (36.8)	31 (39.7)			
	Highly interesting and fun	79 (33.9)	29 (36.7)	29 (38.2)	21 (26.9)			
**Relevance of intervention**								.59
	Strongly disagree	7 (3)	1 (1.3)	3 (3.9)	3 (3.8)			
	Disagree	16 (6.9)	3 (3.8)	7 (9.2)	6 (7.7)			
	Neither agree nor disagree	40 (17.2)	14 (17.7)	9 (11.8)	17 (21.8)			
	Agree	97 (41.6)	36 (45.6)	33 (43.4)	28 (35.9)			
	Strongly agree	73 (31.3)	25 (31.6)	24 (31.6)	24 (30.8)			
**Satisfaction with intervention**								.71
	Very dissatisfied	3 (1.3)	0 (0)	1 (1.3)	2 (2.6)			
	Dissatisfied	8 (3.4)	2 (2.5)	3 (3.9)	3 (3.8)			
	Neither satisfied nor dissatisfied	19 (8.2)	4 (5.1)	7 (9.2)	8 (10.3)			
	Satisfied	99 (42.5)	36 (45.6)	28 (36.8)	35 (44.9)			
	Very satisfied	104 (44.6)	37 (46.8)	37 (48.7)	30 (38.5)			
**Quality of intervention**								.02
	Poor	5 (2.1)	0 (0)	3 (3.9)	2 (2.6)			
	Okay	25 (10.7)	4 (5.1)	9 (11.8)	12 (15.4)			
	Good	96 (41.2)	42 (53.2)	23 (30.3)	31 (39.7)			
	Excellent	107 (45.9)	33 (41.8)	41 (53.9)	33 (42.3)			
**Likelihood of recommending intervention**								.13
	I would not recommend it to anyone	7 (3)	1 (1.3)	2 (2.6)	4 (5.1)			
	There are very few people I would recommend it to	29 (12.4)	5 (6.3)	11 (14.5)	13 (16.7)			
	There are several people whom I would recommend it to	65 (27.9)	28 (35.4)	17 (22.4)	20 (25.6)			
	There are many people I would recommend it to	74 (31.8)	20 (25.3)	29 (38.2)	25 (32.1)			
	Definitely—I would recommend it to everyone	58 (24.9)	25 (31.6)	17 (22.4)	16 (20.5)			
**Ease of use of *Unmind* app and intervention**								.96
	No (limited instructions, confusing, and complicated)	1 (0.4)	0 (0)	0 (0)	1 (1.3)			
	Useable after a lot of time and effort	1 (0.4)	0 (0)	1 (1.3)	0 (0)			
	Usable after some time and effort	12 (5.2)	4 (5.1)	4 (5.3)	4 (5.1)			
	Easy to learn how to use	75 (32.2)	26 (32.9)	22 (28.9)	27 (34.6)			
	Able to use app immediately	144 (61.8)	49 (62)	49 (64.5)	46 (59)			
**Negative effects during the intervention**								.11
	Yes	2 (0.9)	0 (0)	2 (2.6)	0 (0)			
	No	231 (99.1)	79 (100)	74 (97.4)	78 (100)			

^a^Fisher exact tests comparing ratings across study arms.

^b^CS: combatting stress.

^c^WW: working with worry.

^d^BR: building resilience.

##### Negative Effects

Of the 76 participants in the *working with worry* arm, 2 (3%) reported experiencing negative effects while completing their intervention. Qualitative feedback suggested that in both instances, this referred to frustration with the intervention (not understanding the content or not finding it useful). Table 4 shows the proportion of participants whose self-reported outcome scores reliably deteriorated from t0 to t1 and t1 to t2 for each study arm and each secondary outcome measure. Across all outcomes, the proportion of participants who self-reported reliable deterioration ranged from 1.1% to 8.9% for t0 to t1 and 2.4% to 12.4% for t1 to t2 (during which participants no longer had access to any interventions). These rates were largely equivalent between the intervention arms and the control group and are consistent with previous estimates that 5% to 10% of participants are expected to deteriorate following in-person psychotherapy [60]. Thus, the interventions in this study did not appear to be associated with symptom deterioration or other unwanted negative effects.

**Table 4:** Number and percentage of participants per study arm (and overall) that reliably deteriorated from time point 0 (t0) to time point 1 (t1) and t1 to time point 2 (t2) based on reliable change index for each secondary outcome measure.

Outcome			Overall, n (%)		Study arm, n (%)						
					Control		CS^a^		WW^b^		BR^c^
**PSS^d^**											
	t1 to t0 (n=365)	15 (4.1)		4 (4.3)		8 (8.9)		1 (1.1)		2 (2.2)	
	t2 to t1 (n=349)	35 (10)		9 (9.9)		6 (7.1)		11 (12.4)		9 (10.7)	
**GAD-7^e^**											
	t1 to t0 (n=353)	18 (5.1)		4 (4.4)		7 (8.0)		1 (1.1)		6 (6.9)	
	t2 to t1 (n=340)	27 (7.9)		9 (10.2)		3 (3.6)		7 (8.4)		8 (9.8)	
**PHQ-8^f^**											
	t1 to t0 (n=359)	17 (4.7)		7 (7.5)		6 (6.7)		1 (1.1)		3 (3.4)	
	t2 to t1 (n=344)	13 (3.8)		3 (3.4)		2 (2.4)		4 (4.5)		4 (4.8)	
**BRS^g^**											
	t1 to t0 (n=356)	14 (3.9)		4 (4.4)		3 (3.4)		4 (4.4)		3 (3.5)	
	t2 to t1 (n=347)	22 (6.3)		5 (5.4)		6 (7.1)		7 (7.9)		4 (4.9)	

^a^CS: combatting stress.

^b^WW: working with worry.

^c^BR: building resilience.

^d^PSS: Perceived Stress Scale.

^e^GAD-7: Generalized Anxiety Disorder-7.

^f^PHQ-8: Patient Health Questionnaire-8.

^g^BRS: Brief Resilience Scale.

### Secondary Outcomes

#### ITT Analyses

##### Overview

The following are based on ITT analyses that include data from all randomized participants (except for those not meeting the eligibility criteria; see Multimedia Appendix 4 for PP results). EMMs for each secondary outcome (grouped by study arm and time point) are shown in Table 5. All study arms reported significant within-group improvements from t0 to t1 on all secondary outcomes (all *P*<.05), except for symptoms of depression (PHQ-8) and resilience (BRS) in the control group.

**Table 5:** Estimated marginal means (EMMs) from linear mixed-effects models at each of the 3 study time points shown for each intervention arm and each secondary outcome measure.

Outcome		Time point		
		Baseline (t0)	Postintervention (t1)	Follow-up (t2)
		EMM (SE; 95% CI)	EMM (SE; 95% CI)	EMM (SE; 95% CI)
**PSS^a^**				
	Control	16.86 (0.76; 15.37-18.35)	15.19^b^ (0.76; 13.69-16.69)	14.62^c^ (0.76; 13.12-16.12)
	CS^d^	16.35 (0.76; 14.86-17.84)	13.30^c^ (0.77; 11.79-14.80)	12.59^c^ (0.77; 11.08-14.10)
	WW^e^	17.14 (0.75; 15.68-18.61)	12.93^c^ (0.76; 11.44-14.42)	12.73^c^ (0.76; 11.24-14.22)
	BR^f^	17.76 (0.74; 16.29-19.22)	13.49^c^ (0.76; 12.01-14.98)	13.17^c^ (0.77; 11.66-14.68)
**GAD-7^g^**				
	Control	6.38 (0.50; 5.41-7.36)	5.26^b^ (0.50; 4.28-6.24)	5.42^h^ (0.50; 4.44-6.40)
	CS	6.20 (0.50; 5.23-7.18)	4.53^c^ (0.50; 3.54-5.52)	4.01^c^ (0.51; 3.02-5.00)
	WW	6.57 (0.49; 5.61-7.53)	4.12^c^ (0.50; 3.15-5.10)	4.12^c^ (0.50; 3.15-5.10)
	BR	7.02 (0.49; 6.06-7.98)	4.69^c^ (0.50; 3.72-5.66)	4.42^c^ (0.51; 3.43-5.41)
**PHQ-8^i^**				
	Control	6.62 (0.51; 5.61-7.63)	6.38^j^ (0.52; 5.37-7.39)	6.06^j^ (0.52; 5.04-7.08)
	CS	6.68 (0.51; 5.67-7.69)	4.94^c^ (0.52; 3.92-5.96)	4.56^c^ (0.52; 3.53-5.59)
	WW	6.85 (0.51; 5.85-7.84)	4.36^c^ (0.51; 3.35-5.36)	4.56^c^ (0.51; 3.55-5.57)
	BR	7.34 (0.50; 6.35-8.33)	5.53^c^ (0.51; 4.53-6.54)	5.33^c^ (0.52; 4.31-6.36)
**BRS^k^**				
	Control	20.37 (0.52; 19.36-21.38)	20.90^j^ (0.52; 19.89-21.92)	21.18^h^ (0.52; 20.17-22.20)
	CS	19.63 (0.52; 18.62-20.64)	21.03^c^ (0.52; 20.01-22.05)	21.30^c^ (0.52; 20.27-22.33)
	WW	19.00 (0.51; 18.00-20.00)	20.70^c^ (0.51; 19.70-21.71)	20.85^c^ (0.51; 19.84-21.86)
	BR	19.05 (0.50; 18.06-20.04)	20.52^c^ (0.51; 19.51-21.52)	21.29^c^ (0.52; 20.27-22.32)

^a^PSS: Perceived Stress Scale.

^b^*P*≤.01; denotes significance of within-group contrasts comparing t0 to t1 and t0 to t2 for each outcome (*P* values are unadjusted).

^c^*P*≤.001; denotes significance of within-group contrasts comparing t0 to t1 and t0 to t2 for each outcome (*P* values are unadjusted).

^d^CS: combatting stress.

^e^WW: working with worry.

^f^BR: building resilience.

^g^GAD-7: Generalized Anxiety Disorder-7.

^h^*P*<.05; denotes significance of within-group contrasts comparing t0 to t1 and t0 to t2 for each outcome (*P* values are unadjusted).

^i^PHQ-8: Patient Health Questionnaire-8.

^j^*P*>.05.

^k^BRS: Brief Resilience Scale.

##### Combatting Stress

Participants in the *combatting stress* arm reported a trend toward larger decreases in perceived stress (t1−t0: Hedges *g*=0.24, 95% CI −0.05 to 0.53; t2−t0: Hedges *g*=0.27, 95% CI −0.02 to 0.56) and larger increases in resilience (t1−t0: Hedges *g*=0.24, 95% CI −0.05 to 0.53; t2−t0: Hedges *g*=0.24, 95% CI −0.05 to 0.53) at both time points; however, none reached significance at an α level of .05 in this sample. Participants also reported greater improvement in symptoms of depression at t1 and t2 (t1−t0*:* Hedges *g=*0.37, 95% CI 0.08-0.66, *P*=.01; t2−t0: Hedges *g*=0.38, 95% CI 0.09-0.67, *P*=.009) and in symptoms of anxiety at t2 (Hedges *g*=0.30, 95% CI 0.01-0.59; *P*=.04) than the control group (Table 6).

**Table 6:** Contrasts and between-group (intervention versus control) effect size calculations from linear mixed-effects models (LMMs) applied to each secondary outcome for both intention-to-treat (ITT) and subgroup analyses.

Outcome				Estimate^a^ (SE; 95% CI)		*P* value				Hedges *g* (95% CI)		
						LMM^b^		Tukey adjusted^c^		ITT		Subgroup
**PSS^d,e^**												
	**t1^f^ minus t0^g^**											
		CS^h^	1.38 (0.83; −0.57 to 3.33)		.10		.34		0.24 (−0.05 to 0.53)		0.29 (−0.08 to 0.66)	
		WW^i^	2.54 (0.82; 0.93 to 4.16)		.002		.01		0.45 (0.16 to 0.74)		0.66 (0.28 to 1.04)	
		BR^j^	2.59 (0.82; 0.97 to 4.21)		.002		.009		0.45 (0.17 to 0.74)		0.47 (0.10 to 0.84)	
		All^k^	2.18 (0.67; 0.86 to 3.50)		.001		.004		0.38 (0.18 to 0.58)		0.47 (0.21 to 0.73)	
	**t2^l^ minus t0**											
		CS	1.52 (0.83; −0.11 to 3.16)		.07		.26		0.27 (−0.02 to 0.56)		0.41 (0.04 to 0.78)	
		WW	2.18 (0.83; 0.55 to 3.80)		.009		.04		0.38 (0.09 to 0.67)		0.64 (0.26 to 1.02)	
		BR	2.34 (0.84; 0.70 to 3.98)		.005		.03		0.40 (0.12 to 0.69)		0.53 (0.16 to 0.90)	
		All	2.02 (0.68; 0.69 to 3.34)		.003		.008		0.35 (0.15 to 0.55)		0.52 (0.26 to 0.78)	
**GAD-7^m,n^**												
	**t0 minus t1**											
		CS	0.55 (0.59; −0.61 to 1.71)		.35		.79		0.14 (−0.15 to 0.42)		0.29 (−0.09 to 0.66)	
		WW	1.32 (0.59; 0.17 to 2.48)		.02		.11		0.33 (0.04 to 0.61)		0.46 (0.09 to 0.84)	
		BR	1.21 (0.59; 0.06 to 2.36)		.04		.17		0.30 (0.01 to 0.58)		0.41 (0.04 to 0.78)	
		All	1.03 (0.48; 0.09 to 1.97)		.03		.08		0.25 (0.05 to 0.46)		0.39 (0.12 to 0.65)	
	**t0 minus t2**											
		CS	1.23 (0.59; 0.06 to 2.40)		.04		.16		0.30 (0.01 to 0.59)		0.49 (0.11 to 0.88)	
		WW	1.48 (0.59; 0.33 to 2.64)		.01		.06		0.36 (0.08 to 0.65)		0.64 (0.25 to 1.02)	
		BR	1.64 (0.60; 0.47 to 2.81)		.006		.03		0.40 (0.11 to 0.68)		0.58 (0.20 to 0.96)	
		All	1.45 (0.48; 0.51 to 2.40)		.003		.008		0.35 (0.15 to 0.56)		0.57 (0.30 to 0.84)	
**PHQ-8^o,p^**												
	**t0 minus t1**											
		CS	1.51 (0.59; 0.34 to 2.67)		.01		.06		0.37 (0.08 to 0.66)		0.47 (0.08 to 0.85)	
		WW	2.25 (0.59; 1.09 to 3.41)		<.001		.001		0.55 (0.26 to 0.84)		0.70 (0.32 to 1.09)	
		BR	1.57 (0.59; 0.41 to 2.73)		.008		.04		0.38 (0.10 to 0.67)		0.46 (0.08 to 0.83)	
		All	1.78 (0.48; 0.83 to 2.72)		<.001		<.001		0.43 (0.23 to 0.64)		0.54 (0.28 to 0.81)	
	**t0 minus t2**											
		CS	1.56 (0.60; 0.39 to 2.74)		.009		.045		0.38 (0.09 to 0.67)		0.57 (0.19 to 0.96)	
		WW	1.72 (0.59; 0.56 to 2.89)		.004		.02		0.42 (0.13 to 0.71)		0.62 (0.24 to 1.00)	
		BR	1.44 (0.60; 0.27 to 2.62)		.02		.08		0.35 (0.06 to 0.63)		0.46 (0.09 to 0.84)	
		All	1.58 (0.49; 0.63 to 2.53)		.001		.003		0.38 (0.18 to 0.59)		0.55 (0.28 to 0.82)	
**BRS^q,r^**												
	**t0 minus t1**											
		CS	0.87 (0.52; −0.15 to 1.90)		.10		.34		0.24 (−0.05 to 0.53)		0.35 (0.16 to 0.85)	
		WW	1.18 (0.52; 0.16 to 2.19)		.02		.11		0.33 (0.04 to 0.62)		0.66 (0.19 to 1.13)	
		BR	0.94 (0.52; −0.08 to 1.96)		.07		.27		0.26 (−0.02 to 0.55)		0.44 (−0.06 to 0.93)	
		All	1.00 (0.42; 0.17 to 1.83)		.02		.049		0.28 (0.08 to 0.48)		0.50 (0.16 to 0.83)	
	**t0 minus t2**											
		CS	0.86 (0.53; −0.17 to 1.89)		.10		.36		0.24 (−0.05 to 0.53)		0.18 (−0.32 to 0.68)	
		WW	1.04 (0.52; 0.01 to 2.06)		.047		.19		0.29 (0.00 to 0.58)		0.47 (0.00 to 0.94)	
		BR	1.43 (0.53; 0.40 to 2.47)		.006		.03		0.39 (0.11 to 0.68)		0.38 (−0.11 to 0.87)	
		All	1.11 (0.43; 0.27 to 1.94)		.01		.03		0.30 (0.10 to 0.51)		0.36 (0.02 to 0.69)	

^a^Contrast estimates from LMMs.

^b^*P* value from LMM (group×time interaction terms).

^c^*P* value following Tukey adjustment for all pairwise comparisons (only comparisons of interest are shown).

^d^PSS: Perceived Stress Scale.

^e^Sample size for ITT analyses n=383; sample size for subgroup analyses n=236.

^f^t1: time point 1.

^g^t0: time point 0.

^h^CS: combatting stress.

^i^WW: working with worry.

^j^BR: building resilience.

^k^Pooled effect of all intervention arms.

^l^t2: time point 2.

^m^GAD-7: Generalized Anxiety Disorder-7.

^n^Sample size for ITT analyses n=383; sample size for subgroup analyses n=227.

^o^PHQ-8: Patient Health Questionnaire-8.

^p^Sample size for ITT analyses n=383; sample size for subgroup analyses n=229.

^q^BRS: Brief Resilience Scale.

^r^Sample size for ITT analyses n=383; sample size for subgroup analyses n=142.

##### Working With Worry

Participants in the *working with worry* arm reported greater improvements in symptoms of anxiety at t1 and t2 than the control group (t1 minus t0: Hedges *g=*0.33, 95% CI 0.04-0.61, *P*=.02; t2 minus t0: Hedges *g*=0.36, 95% CI 0.08-0.65, *P*=.01), as well as greater improvements across all other secondary outcomes (all *P*<.05; Hedges *g* range 0.33-0.55; Table 6). All improvements were maintained at t2 (all *P*<.05; Hedges *g* range 0.29-0.42). Effect sizes were largest for symptoms of depression at both t1 (Hedges *g*=0.55, 95% CI 0.26-0.84) and t2 (Hedges *g*=0.42, 95% CI 0.13-0.71).

##### Building Resilience

Participants in the *building resilience* arm reported a trend toward larger increases in resilience at t1 than the control group (t1 minus t0: Hedges *g*=0.26, 95% CI −0.02 to 0.55), which emerged as significant at t2 (t2 minus t0: Hedges *g*=0.39, 95% CI 0.11-0.68; *P*=.006). In addition, participants reported significantly greater improvements across all other secondary outcomes at t1 (all *P*<.05; Hedges *g* range 0.30-0.45), which were maintained at t2 (all *P*<.05; Hedges *g* range 0.35-0.40; Table 6).

A comparison of the overall (pooled) effect of all intervention arms relative to the control group revealed significantly greater improvement for all 4 secondary outcome measures (Hedges *g* range 0.25−0.43; Table 6). In addition, post hoc contrasts on the LMM estimates suggested that none of the intervention arms were significantly different from one another when comparing t0 to t1 or t2 for any of the secondary outcome measures (all *P*>.05), although this study was not powered to detect such differences. Finally, when comparing score changes from t1 to t2, there were no significant differences between the control group and any of the intervention arms for any of the secondary outcome measures (all *P*>.05).

##### Subgroup Analyses

Subgroup analyses were performed for all secondary outcome measures to explore intervention effects for participants self-reporting at least mild symptoms (or moderately low resilience) at t0 (see Multimedia Appendix 7 for subgroup sample sizes and baseline scores). Findings were largely equivalent to the ITT analysis, although between-group effect sizes were generally larger, ranging from 0.39 (95% CI 0.12-0.65) to 0.54 (95% CI 0.28-0.81) when pooling the intervention arms (Table 6).

##### Exploratory Analyses

Exploratory multiple logistic regression suggested that participants working in health care (b=−2.11, SE 0.86; *P*=.01), finance, insurance, or real estate (b=−2.57, SE 0.88; *P*=.004), and professional services (b=−1.87, SE 0.91; *P*=.04) were less likely to complete their allocated intervention relative to those working in industrials (the reference category). The completion rate for industrials was 83.3% compared with 51.9% for health care, 41.2% for finance, insurance, or real estate, and 55.6% for professional services.

In addition, participants with higher PHQ-8 scores at baseline were less likely to complete their intervention (b=−0.17, SE 0.05; *P*<.001). None of the demographic variables collected in this study predicted completion (all *P*>.05). These analyses also confirmed that participants allocated to *combatting stress* were more likely to complete their intervention than those allocated to *building resilience* (b=−1.05, SE 0.37; *P*=.005) while controlling for all other baseline variables.

## Discussion

### Principal Findings

Intervention research can be undermined by problems with intervention delivery, acceptability, participant retention, and smaller than anticipated effect sizes. Therefore, guidelines suggest conducting pilot studies to test trial feasibility and estimate important trial parameters before running a definitive trial [61]. This study reports on the feasibility and preliminary efficacy of 3 interventions featured on the *Unmind* MHapp when delivered to working adults in the United Kingdom. The study methods and interventions were found to be feasible, and all preregistered progression criteria were met. This suggests that a definitive trial is warranted, although several minor proposed protocol amendments are discussed.

Participant retention and intervention adherence were largely consistent with or higher than those in comparable studies. For instance, only 7% (27/383) of participants were lost to attrition at follow-up, which compares favorably with recent meta-analyses reporting average attrition rates of 23% to 48% for MHapp trials [11,16,62]. This may be because of the use of the Prolific recruitment platform, which is associated with high participant response rates [35], and reimbursing participants at each study assessment. It may also suggest that participants, on average, perceived the *Unmind* platform as helpful and engaging and were thus motivated to complete the study.

Objective engagement data suggested that 67.8% (196/289) of randomized participants (and 196/237, 82.7% of those starting their intervention) completed all intervention sessions, which is similar to or higher than average completion rates ranging between 30% and 65% for other MHapp interventions [11,16,53,63]. These engagement rates are particularly encouraging, considering that participants were not recruited on the basis of seeking help for a mental health problem and were not randomized to an intervention based on scoring poorly on the target outcome at baseline.

Despite these high levels of engagement, the study interventions were brief, comprising approximately 60 to 80 minutes of learning over 7 sessions. In addition, the study used a nonclinical sample, and regression analyses suggested that participants with higher symptom severity at baseline were less likely to complete their allocated intervention. Thus, these findings may not be generalizable to other interventions featured on the *Unmind* app or to other study populations. Future studies designed to evaluate the use of the *Unmind* app in subclinical populations are currently being planned. Interestingly, although participants with higher baseline symptoms were less likely to complete their intervention, feedback ratings at postintervention were largely equivalent across the study sample. For example, 87.1% (203/233) of all participants reported being either *satisfied* or *very satisfied* with their intervention and rated the quality of their intervention as either *good* or *excellent*, and these ratings did not differ for participants with more severe symptoms at baseline.

Although all 3 interventions met progression criteria, participants randomized to the *building resilience* arm were marginally less likely to start their allocated intervention and complete all intervention sessions after starting. A potential explanation is that participants may have felt less motivated by the theme of resilience as compared with stress and anxiety. In addition, participants in the *building resilience* arm reported marginally worse mental health scores at baseline, which may have negatively affected engagement. Although participant feedback was largely equivalent across the 3 interventions, there were several marginal (not statistically significant) differences that may be a contributing factor. For example, compared with *combatting stress*, participants in the *building resilience* arm were slightly less likely to rate the intervention as *good* relative to *okay* or *poor*. Although it is difficult to draw firm conclusions from these data, it will be important to test whether such differences persist in a definitive trial and the extent to which any changes or improvements to the *building resilience* intervention are warranted.

The findings from this pilot study revealed several opportunities for minor protocol improvements before running a definitive trial. First, of the 383 participants, 11 (2.9%) reported not fully understanding the study instructions, and 1.7% (5/289) of participants randomized to an intervention engaged with the wrong intervention. This could be addressed by providing participants with detailed video instructions and implementing a brief quiz to ensure that all participants understand how to access their allocated intervention. If feasible, participants could be given access to a modified, study-specific version of the *Unmind* platform that only includes the interventions being tested. Second, discrepancies between self-reported and objective in-app engagement meant that 48% (45/93) of participants who stopped using the *Unmind* app did not provide feedback on their reasons for discontinuing their intervention. This could be addressed by restructuring the feedback questionnaire to capture data from all participants, regardless of self-reported engagement. Third, 0.9% (2/233) of participants reported experiencing negative effects while completing their intervention; however, qualitative feedback suggested that both instances referred to frustration with the intervention (not understanding the content or not finding it useful). This question could be modified to capture *lasting* bad effects (refer to the study by Crawford et al [64]) with more specific clarifying questions to better differentiate harmful effects from frustration and/or lack of intervention enjoyment. Finally, 4% (16/400) of participants were excluded from the analysis as they reported being unemployed at baseline, which was in contrast to their prescreening responses. Although the Prolific platform precludes the use of additional screening questions at baseline, the sample size calculation for a definitive trial could be adjusted to account for this potential discrepancy.

Although the study was not powered for formal hypothesis testing, preliminary efficacy findings suggested that the study interventions were associated with small to moderate between-group improvements in ≥1 mental health outcomes, which were maintained at the 1-month follow-up. This is consistent with findings from meta-analyses of previous MHapp trials [11,12,26]. All 3 interventions resulted in larger improvements in symptoms of depression, anxiety, perceived stress, and resilience than the control group, although smaller effects (Hedges *g*<0.3) did not reach statistical significance. An appropriately powered definitive trial may be more likely to capture such small effects with greater precision and, where possible, should aim to report on the clinical importance of these findings (eg, with regard to minimal important difference thresholds). These efficacy findings were consistent across both PP and ITT analyses and are particularly promising, given the brevity of the interventions. In addition, the findings were robust across subgroup analyses that only included participants with at least mild problems at baseline. However, 2 further patterns emerged that merit discussion.

First, relative to baseline, participants in the control group reported statistically significant improvements in stress and anxiety at both study time points and improvements in resilience at the 1-month follow-up, despite not having access to any study interventions. Although this may reflect phenomena such as practice effects or regression to the mean, it is worth noting that baseline data were collected several weeks after the commencement of a third national UK lockdown (in response to rising cases of SARS-CoV-2), whereas follow-up data were collected after the start of a phased easing of restrictions. A longitudinal survey conducted in England suggests that symptoms of anxiety and depression tend to rise rapidly during the early stages of a lockdown but decline quickly thereafter [65], which may partly explain the changes in mental health scores reported by the control group. Thus, the present efficacy findings may not be directly generalizable to other contexts. Importantly, the intervention groups reported larger improvements in mental health despite significant changes in the control group, and MHapps such as *Unmind* may be an effective way of delivering accessible mental health support to workforces working remotely or during times of national crisis.

Second, although all 3 interventions resulted in significant improvements for at least one mental health outcome, the study interventions did not appear to be sensitive or specific to their target outcome. For example, the *combatting stress* arm resulted in small between-group (intervention vs control) improvements in perceived stress (not statistically significant), as well as significant reductions in symptoms of depression. Similarly, participants in the *working with worry* arm reported significant improvements across all outcomes, despite the intervention specifically targeting anxiety. A potential explanation for this is that different facets of mental health tend to strongly covary with one another [66], and transdiagnostic research suggests that symptoms of different mental health problems often respond to the same treatments [67]. The present findings lend credence to this, as the study interventions all primarily involve similar CBT-based techniques (identifying and challenging negative thinking patterns, problem solving, and breaking negative cycles of thinking or behaviors). As this study was not powered to detect differences between intervention arms (or to test whether effect sizes for some outcomes were significantly different from others), it is not possible to draw meaningful conclusions from these data. Future studies should aim to test for any such differences so that users of the *Unmind* app can access interventions that are most likely to benefit their individual problem areas.

### Strengths and Limitations

This study had several strengths. First, intervention adherence and engagement were objectively captured via *Unmind* (the intervention platform). This is important, given the recent evidence of substantial discrepancies between self-reported and objective adherence in digital interventions [68]. Second, participant retention was very high, which is extremely important as missing data can reduce statistical power and lead to biased intervention effects. Second, all study outcomes and analyses were prospectively registered, precluding any selective reporting of outcomes or nonpublication of findings [69]. Third, the study recruited a sample representative of the general UK population with respect to age, sex, and ethnicity, and participants were employed across a variety of industries. This is important as *Unmind* is designed for use across a broad range of demographics, and a lack of diversity within study samples can limit the generalizability of the study findings.

A limitation of this study is the use of a passive no-intervention control group, as opposed to an active control group in which participants engage with activities matched for duration, attention, and interest. Passive controls do not allow true intervention effects to be differentiated from nonspecific placebo effects and may introduce nocebo effects [31]. Although it is useful in practical terms to estimate this combined effect of the *Unmind* app, active controls are needed to fully understand the mechanisms underlying its effects. In addition, despite being randomly assigned, participants in the control group had slightly higher levels of self-reported resilience at baseline, and it is unknown whether this may have affected between-group differences in resilience scores over time. In addition, as with most web-based trials, participants were not blinded to group allocation. Finally, although the Prolific recruitment platform has several strengths, the participant pool was entirely self-selected, and it remains unknown to what extent the present findings are generalizable to working adults nationwide.

### Conclusions

This study assessed the feasibility of conducting a future definitive RCT to evaluate the efficacy of 3 brief interventions featured on the *Unmind* MHapp. The study methods and interventions were found to be feasible, and all preregistered criteria for progression to a definitive trial were met. Several minor protocol amendments have been suggested. Preliminary efficacy findings indicate that the study interventions may result in improved mental health outcomes when offered to working adults.

## Appendix

Multimedia Appendix 1 Postintervention feedback questions.

Multimedia Appendix 2 Deviations from the preregistered data analysis plan.

Multimedia Appendix 3 Reliable change index calculations.

Multimedia Appendix 4 Per-protocol analyses.

Multimedia Appendix 5 Postintervention feedback ratings for subgroups.

Multimedia Appendix 6 Participants’ self-reported reasons for not starting or discontinuing their intervention.

Multimedia Appendix 7 Subgroup sample sizes and baseline scores.

Multimedia Appendix 8 CONSORT-eHEALTH checklist (V 1.6.1).

## Abbreviations

ACT: acceptance and commitment therapy
BRS: Brief Resilience Scale
CBT: cognitive behavioral therapy
CONSORT: Consolidated Standards of Reporting Trials
EMM: estimated marginal mean
GAD: generalized anxiety disorder
ITT: intention-to-treat
LMM: linear mixed-effects model
MHapp: mental health app
MM: mindfulness meditation
PHQ: Patient Health Questionnaire
PP: per-protocol
PSS: Perceived Stress Scale
RCT: randomized controlled trial
t0: time point 0
t1: time point 1
t2: time point 2
