# Supplementary material for: Feasibility and Preliminary Efficacy of Web-Based and Mobile Interventions for Common Mental Health Problems in Working Adults: Multi-Arm Randomized Pilot Trial
Source: JMIR Form Res. 2022 Mar 3;6(3):e34032. doi: 10.2196/34032 (PMC8931651; doi:10.2196/34032)
Supplement: Multimedia Appendix 1 [file formative_v6i3e34032_app1.docx]

# **Multimedia Appendix 1**

Post-intervention feedback questions

1. Thinking about the design of the Unmind Series, did you find it interesting or fun to engage with? (Adapted from Section A of the MARS)
   1. Dull, not fun or interesting at all
   2. Mostly boring
   3. OK, fun enough to entertain me for a brief time
   4. Moderately interesting and fun, would entertain me for some time
   5. Highly interesting and fun
2. Thinking about the content in the Unmind Series, did you find it interesting or fun to engage with? (Adapted from Section A of the MARS)
   1. Dull, not fun or interesting at all
   2. Mostly boring
   3. OK, fun enough to entertain me for a brief time
   4. Moderately interesting and fun, would entertain me for some time
   5. Highly interesting and fun
3. Did you find the Unmind app and Series easy to use? (Adapted from Section B of the MARS)
   1. No (limited instructions; confusing menu labels/icons; complicated)
   2. Useable after a lot of time and effort
   3. Useable after some time and effort
   4. Easy to learn how to use the app (or has clear instructions)
   5. Able to use app immediately; intuitive; simple
4. Would you recommend the Unmind Series to people who might benefit from it, such as friends, family, or colleagues?
   1. Not at all – I would not recommend it to anyone
   2. There are very few people I would recommend it to
   3. Maybe – There are several people whom I would recommend it to
   4. There are many people I would recommend it to
   5. Definitely – I would recommend it to everyone
5. Would you agree that the Unmind Series was relevant to your personal experience?
   1. Strongly Disagree
   2. Disagree
   3. Neither Agree nor Disagree
   4. Agree
   5. Strongly Agree
6. Did you experience any negative effects whilst completing the Unmind Series?
   1. Yes
   2. No
7. How satisfied are you with the Unmind Series you were asked to complete overall?
   1. Very Dissatisfied
   2. Dissatisfied
   3. Neither Satisfied nor Dissatisfied
   4. Satisfied
   5. Very Satisfied
8. How would you rate the quality of the Unmind Series you were asked to complete?
   1. Poor
   2. Okay
   3. Good
   4. Excellent
