# Supplementary material for: Feasibility and Preliminary Efficacy of Web-Based and Mobile Interventions for Common Mental Health Problems in Working Adults: Multi-Arm Randomized Pilot Trial
Source: JMIR Form Res. 2022 Mar 3;6(3):e34032. doi: 10.2196/34032 (PMC8931651; doi:10.2196/34032)
Supplement: Multimedia Appendix 2 [file formative_v6i3e34032_app2.docx]

# **Multimedia Appendix 2**

Deviations from the preregistered data analysis plan

Several minor deviations were made from the preregistered data analysis plan to streamline analyses, simplify the reporting of results, and provide additional statistical comparisons between the three intervention arms for relevant primary outcomes. These are detailed below.

1. In the study protocol, we defined the cutoff for inclusion in PSS subgroup analyses as a score ≥13 at baseline, based on normative data from original validation work [42]. However, more recent findings suggest that average stress levels in the general population have substantially increased [60]. We therefore adjusted the cutoff to ≥16, to match current normative data.
2. The study protocol specified that subgroup analyses would be performed for *all* primary outcomes. However, intervention uptake, adherence to intervention instructions, engagement, and retention were all high in the overall sample, with insufficient variance to warrant subgroup analyses. In addition, we explored the impact of baseline symptom severity on intervention engagement via regression analyses, which were considered more sensitive than applying descriptive statistics to subgroups. Post-intervention feedback was largely equivalent when comparing ratings from subgroups to the overall sample, and thus subgroup ratings are reported in the Multimedia Appendix only.
3. Fisher’s exact test of independence was used to compare categorical responses (such as post-intervention ratings) between intervention arms, to better understand potential differences between intervention arms across primary outcomes.
4. In the study protocol, we specified that the PP analysis would exclude participants who engaged with more than one session outside of their allocated intervention arm. However, since most participants did not stray from their allocated intervention, we opted for a simpler and more conservative approach of excluding participants who engaged with *any* sessions outside of their intervention arm. Participants who were lost to follow-up at *t1* and *t2* were also excluded. We opted to report the results of the PP analysis in the Multimedia Appendix only, as these were largely equivalent to the more conservative ITT approach.
5. To simplify the reporting of results, we opted not to compute within-group Hedge’s *g* effect sizes for each intervention arm across the three study timepoints, as these are less informative than between-group effect sizes (which estimate “true” intervention effects). However, *P* value significance for within-group contrasts (*t0* - *t1*, and *t0* - *t2*) are included in Table 5.
6. We omitted logistic regression analyses designed to explore whether any baseline variables were predictive of attrition at *t1* and *t2*, as attrition was much lower than expected and such analyses would have been inappropriate. Similarly, we omitted linear regression analyses designed to explore whether intervention adherence (number of sessions completed) was predictive of change in secondary outcome measures from t*0* to *t1*, as only ~14% of randomized participants discontinued their intervention prior to completing all sessions, and such analyses would have likely been underpowered.
7. In the study protocol, we planned to report on the proportion of participants whose self-reported scores improved, deteriorated, or remained unchanged from *t0*-*t1* for all secondary outcome measures, as a means of identifying any potential negative effects associated with any of the interventions. To make this analysis more streamlined and robust, we opted to focus on deterioration only, and report the number and proportion of participants showing *reliable* deterioration between *t0*-*t1* and *t1*-*t2*, based on a reliable change index (RCI) that was computed for each self-reported outcome measure. RCI accounts for measurement noise, and this approach is more consistent with previously published consensus guidelines on measuring negative effects of digital interventions [41].
8. The secondary outcomes (as specified in the study protocol) included a measure of overall mental health and wellbeing (the Unmind Index). However, since the Unmind Index is newly developed and has not yet been shown to be sensitive/responsive to change, we chose not to analyze these data as part of this pilot study. The data will contribute to further psychometric testing of the Unmind Index and will be reported elsewhere.
9. Participants were able to provide optional, open-ended, qualitative feedback regarding the intervention they were asked to engage with at post-intervention. Initial inspection of this feedback suggested it included a wide variety of topics and themes, requiring formal qualitative methods (such as framework, thematic, or grounded theory-informed analysis) to analyze and interpret. This was considered beyond the scope of this study and the skillset of the research team and was thus omitted from this paper. These data may be analyzed and reported in a future paper describing qualitative findings from the Unmind app.
