# Supplementary material for: Feasibility and Preliminary Efficacy of Web-Based and Mobile Interventions for Common Mental Health Problems in Working Adults: Multi-Arm Randomized Pilot Trial
Source: JMIR Form Res. 2022 Mar 3;6(3):e34032. doi: 10.2196/34032 (PMC8931651; doi:10.2196/34032)
Supplement: Multimedia Appendix 3 [file formative_v6i3e34032_app3.docx]

# **Multimedia Appendix 3**

Reliable change index (RCI) calculations (for each secondary outcome measure).

| Outcome | Cronbach’s  alpha | Reliable change index (RCI) | Reliable change criterion (RCC) |
| --- | --- | --- | --- |
| **PSS** | 0.91 | 3.23 | 6.33 |
| **GAD-7** | 0.91 | 2.19 | 4.29 |
| **PHQ-8** | 0.88 | 2.61 | 5.11 |
| **BRS** | 0.91 | 2.16 | 4.22 |
