# Supplementary material for: Feasibility and Preliminary Efficacy of Web-Based and Mobile Interventions for Common Mental Health Problems in Working Adults: Multi-Arm Randomized Pilot Trial
Source: JMIR Form Res. 2022 Mar 3;6(3):e34032. doi: 10.2196/34032 (PMC8931651; doi:10.2196/34032)
Supplement: Multimedia Appendix 4 [file formative_v6i3e34032_app4.docx]

# **Multimedia Appendix 4**

Per protocol (PP) contrasts and between-group (intervention vs control) effect size calculations from LMMs applied to each secondary outcome (n = 285). Est = contrast estimates from LMMs; SE = standard error; *P^1^ = P* value from LMM (group x time interaction terms); *P^2^* = *P* value following Tukey adjustment for all pairwise comparisons (only comparisons of interest are shown); *g^PP^* = between-group Hedge’s *g* effect size for subgroups; *CS* = Combatting Stress; *WW* = Working With Worry; *BR* = Building Resilience; All = pooled effect of all intervention arms.

| Outcome | Est [95% CI] | | | SE | *P*^1^ | *P*^2^ | *g^PP^* [95% CI] | | |
| --- | --- | --- | --- | --- | --- | --- | --- | --- | --- |
| **PSS** | | | | | | |  | | |
| *t1 - t0* |  | | |  |  |  |  | | |
| *CS* | 1.32 [0.40 3.03] | | | 0.87 | .13 | .43 | 0.22 [-0.07 0.51] | | |
| *WW* | 3.76 [1.96 5.56] | | | 0.92 | <.001 | <.001 | 0.59 [0.30 0.88] | | |
| *BR* | 3.06 [1.22 4.91] | | | 0.94 | .001 | .007 | 0.47 [0.18 0.76] | | |
| All | 2.63 [1.23 4.02] | | | 0.71 | <.001 | <.001 | 0.40 [0.20 0.60] | | |
| *t2 - t0* |  |  |  |  |  |  |  | | |
| *CS* | 1.62 [-0.11 3.35] | | | 0.88 | .07 | .26 | 0.27 [-0.02 0.56] | | |
| *WW* | 3.12 [1.29 4.94] | | | 0.93 | <.001 | .005 | 0.48 [0.20 0.77] | | |
| *BR* | 2.10 [0.24 3.96] | | | 0.95 | .03 | .12 | 0.32 [0.03 0.60] | | |
| All | 2.24 [0.57 3.92] | | | 0.71 | .002 | .005 | 0.34 [0.13 0.54] | | |
| **GAD-7** | | |  |  |  |  |  | | |
| *t0 - t1* |  |  |  |  |  |  |  | | |
| *CS* | 0.83 [-0.37 2.02] | | | 0.61 | .17 | .52 | 0.20 [-0.09 0.49] | | |
| *WW* | 1.63 [0.38 2.88] | | | 0.64 | .01 | .05 | 0.37 [0.08 0.66] | | |
| *BR* | 1.61 [0.32 2.89] | | | 0.66 | .01 | .07 | 0.35 [0.07 0.64] | | |
| All | 1.32 [0.35 2.28] | | | 0.49 | .007 | .02 | 0.29 [0.09 0.49] | | |
| *t0 - t2* |  |  |  |  |  |  |  | | |
| *CS* | 1.54 [0.33 2.74] | | | 0.62 | .01 | .06 | 0.36 [0.07 0.66] | | |
| *WW* | 2.03 [0.36 3.69] | | | 0.65 | .002 | .01 | 0.45 [0.16 0.74] | | |
| *BR* | 1.49 [0.20 2.79] | | | 0.66 | .02 | .11 | 0.32 [0.04 0.61] | | |
| All | 1.68 [0.71 2.65] | | | 0.50 | <.001 | .002 | 0.36 [0.16 0.56] | | |
| **PHQ-8** | | |  |  |  |  |  | | |
| *t0 - t1* |  |  |  |  |  |  |  | | |
| *CS* | 1.82 [0.57 3.06] | | | 0.63 | .004 | .02 | 0.42 [0.13 0.71] | | |
| *WW* | 2.42 [1.12 3.72] | | | 0.66 | <.001 | .002 | 0.53 [0.24 0.82] | | |
| *BR* | 1.62 [0.28 2.95] | | | 0.68 | .02 | .08 | 0.34 [0.05 0.63] | | |
| All | 1.95 [0.95 2.95] | | | 0.51 | <.001 | <.001 | 0.41 [0.21 0.61] | | |
| *t0 - t2* |  |  |  |  |  |  |  | | |
| *CS* | 1.82 [0.57 3.08] | | | 0.64 | .005 | .02 | 0.42 [0.12 0.71] | | |
| *WW* | 1.94 [0.62 3.25] | | | 0.67 | .004 | .02 | 0.42 [0.13 0.70] | | |
| *BR* | 0.89 [-0.46 2.24] | | | 0.69 | .20 | .57 | 0.19 [-0.10 0.47] | | |
| All | 1.58 [0.57 2.59] | | | 0.52 | .002 | .006 | 0.33 [0.13 0.53] | | |
| **BRS** | | |  |  |  |  |  | | |
| *t0 - t1* |  |  |  |  |  |  |  | | |
| *CS* | 0.98 [-0.06 2.02] | | | 0.53 | .07 | .25 | 0.27 [-0.02 0.56] | | |
| *WW* | 1.63 [0.55 2.72] | | | 0.56 | .003 | .02 | 0.42 [0.14 0.71] | | |
| *BR* | 1.30 [0.18 2.42] | | | 0.57 | .02 | .10 | 0.33 [0.04 0.61] | | |
| All | 1.29 [0.45 2.12] | | | 0.43 | .003 | .008 | 0.32 [0.12 0.52] | | |
| *t0 - t2* |  |  |  |  |  |  |  |  |  |
| *CS* | 0.92 [-0.13 1.97] | | | 0.54 | .09 | .31 | 0.25 [-0.04 0.54] | | |
| *WW* | 1.78 [0.67 2.88] | | | 0.56 | .002 | .009 | 0.46 [0.17 0.74] | | |
| *BR* | 1.67 [0.54 2.80] | | | 0.58 | .004 | .02 | 0.42 [0.13 0.70] | | |
| All | 1.42 [0.57 2.26] | | | 0.43 | .001 | .003 | 0.35 [0.15 0.55] | | |
