# Supplementary material for: Feasibility and Preliminary Efficacy of Web-Based and Mobile Interventions for Common Mental Health Problems in Working Adults: Multi-Arm Randomized Pilot Trial
Source: JMIR Form Res. 2022 Mar 3;6(3):e34032. doi: 10.2196/34032 (PMC8931651; doi:10.2196/34032)
Supplement: Multimedia Appendix 5 [file formative_v6i3e34032_app5.docx]

# **Multimedia Appendix 5**

Post-intervention feedback ratings for participants scoring above or below predefined cutoffs for subgroup analyses at baseline. Ratings are pooled across active intervention arms for each secondary outcome subgroup.

|  | Subgroups | | | |
| --- | --- | --- | --- | --- |
| **Variable** | PSS (n=144) | GAD-7 (n=137) | PHQ-8 (n=137) | BRS (n=88) |
| **Intervention arm** |  |  |  |  |
| CS | 49 (34%) | 46 (34%) | 46 (34%) | 28 (32%) |
| WW | 47 (33%) | 45 (33%) | 47 (34%) | 26 (30%) |
| BR | 48 (33%) | 46 (34%) | 44 (32%) | 34 (39%) |
| **Design of intervention** |  |  |  |  |
| Dull, not fun | 1 (0.7%) | 1 (0.7%) | 1 (0.7%) | 0 (0%) |
| Mostly boring | 8 (5.6%) | 7 (5.1%) | 8 (5.8%) | 3 (3.4%) |
| OK, fun enough | 36 (25%) | 35 (26%) | 34 (25%) | 27 (31%) |
| Moderately interesting & fun | 74 (51%) | 65 (47%) | 67 (49%) | 43 (49%) |
| Highly interesting & fun | 25 (17%) | 29 (21%) | 27 (20%) | 15 (17%) |
| **Content of intervention** |  |  |  |  |
| Dull, not fun | 3 (2.1%) | 3 (2.2%) | 3 (2.2%) | 1 (1.1%) |
| Mostly boring | 4 (2.8%) | 4 (2.9%) | 5 (3.6%) | 3 (3.4%) |
| OK, fun enough | 32 (22%) | 28 (20%) | 29 (21%) | 21 (24%) |
| Moderately interesting & fun | 63 (44%) | 60 (44%) | 58 (42%) | 32 (36%) |
| Highly interesting & fun | 42 (29%) | 42 (31%) | 42 (31%) | 31 (35%) |
| **Relevance of intervention** |  |  |  |  |
| Strongly disagree | 5 (3.5%) | 4 (2.9%) | 4 (2.9%) | 3 (3.4%) |
| Disagree | 7 (4.9%) | 6 (4.4%) | 7 (5.1%) | 4 (4.5%) |
| Neither agree nor disagree | 17 (12%) | 14 (10%) | 18 (13%) | 8 (9.1%) |
| Agree | 68 (47%) | 65 (47%) | 59 (43%) | 42 (48%) |
| Strongly agree | 47 (33%) | 48 (35%) | 49 (36%) | 31 (35%) |
| **Satisfaction with intervention** |  |  |  |  |
| Very dissatisfied | 3 (2.1%) | 3 (2.2%) | 2 (1.5%) | 2 (2.3%) |
| Dissatisfied | 7 (4.9%) | 6 (4.4%) | 7 (5.1%) | 3 (3.4%) |
| Neither satisfied nor dissatisfied | 7 (4.9%) | 4 (2.9%) | 8 (5.8%) | 4 (4.5%) |
| Satisfied | 65 (45%) | 62 (45%) | 57 (42%) | 39 (44%) |
| Very satisfied | 62 (43%) | 62 (45%) | 63 (46%) | 40 (45%) |
| **Quality of intervention** |  |  |  |  |
| Poor | 4 (2.8%) | 4 (2.9%) | 3 (2.2%) | 2 (2.3%) |
| Okay | 14 (9.7%) | 11 (8.0%) | 14 (10%) | 7 (8.0%) |
| Good | 61 (42%) | 58 (42%) | 55 (40%) | 37 (42%) |
| Excellent | 65 (45%) | 64 (47%) | 65 (47%) | 42 (48%) |
| **Likelihood of recommending intervention** |  |  |  |  |
| I would not recommend it to anyone | 6 (4.2%) | 6 (4.4%) | 5 (3.6%) | 4 (4.5%) |
| There are very few people I would recommend it to | 13 (9.0%) | 12 (8.8%) | 14 (10%) | 10 (11%) |
| There are several people whom I would recommend it to | 46 (32%) | 40 (29%) | 43 (31%) | 26 (30%) |
| There are many people I would recommend it to | 45 (31%) | 49 (36%) | 47 (34%) | 29 (33%) |
| Definitely - I would recommend it to everyone | 34 (24%) | 30 (22%) | 28 (20%) | 19 (22%) |
| **Ease of use of Unmind app and intervention** |  |  |  |  |
| No (limited instructions, confusing, complicated) | 1 (0.7%) | 0 (0%) | 1 (0.7%) | 1 (1.1%) |
| Useable after a lot of time and effort | 1 (0.7%) | 1 (0.7%) | 1 (0.7%) | 1 (1.1%) |
| Usable after some time and effort | 9 (6.2%) | 5 (3.6%) | 8 (5.8%) | 4 (4.5%) |
| Easy to learn how to use | 49 (34%) | 46 (34%) | 45 (33%) | 28 (32%) |
| Able to use app immediately | 84 (58%) | 85 (62%) | 82 (60%) | 54 (61%) |
| **Negative effects during intervention** |  |  |  |  |
| Yes | 1 (0.7%) | 1 (0.7%) | 1 (0.7% | 0 (0%) |
| No | 143 (99%) | 136 (99%) | 136 (99%) | 88 (100%) |
