# Supplementary material for: Feasibility and Preliminary Efficacy of Web-Based and Mobile Interventions for Common Mental Health Problems in Working Adults: Multi-Arm Randomized Pilot Trial
Source: JMIR Form Res. 2022 Mar 3;6(3):e34032. doi: 10.2196/34032 (PMC8931651; doi:10.2196/34032)
Supplement: Multimedia Appendix 6 [file formative_v6i3e34032_app6.docx]

# **Multimedia Appendix 6**

Participants’ self-report reasons for not starting and/or discontinuing their allocated intervention. ‘Unknown’ corresponds to participants who were lost to follow-up at *t1*, or who inaccurately reported completing their allocated intervention (despite objective engagement data suggesting the contrary). *CS* = Combatting Stress; *WW* = Working With Worry; *BR* = Building Resilience.

|  | **Overall**  **(n = 93)** | ***CS***  **(n = 20)** | ***WW***  **(n = 34)** | ***BR***  **(n = 39)** |
| --- | --- | --- | --- | --- |
| **Reason for not starting and/or discontinuing the study intervention** | **n** | **n** | **n** | **n** |
| I wasn’t able to create an Unmind account, or the instructions were unclear | 7 | 2 | 3 | 2 |
| I didn’t have time to finish the Series (intervention) | 12 | 2 | 6 | 4 |
| I forgot that I was taking part in the study | 4 | 2 | 1 | 1 |
| I lost motivation to take part in the study | 2 | 0 | 1 | 1 |
| I didn’t enjoy the Unmind Series (intervention) or I found it boring | 2 | 0 | 2 | 0 |
| I didn’t feel like it was helpful in any way | 3 | 0 | 2 | 1 |
| I experienced technical difficulties | 5 | 1 | 2 | 2 |
| I didn’t have access to the Internet | 1 | 0 | 1 | 0 |
| I didn't realize I was supposed to complete any exercises as part of the study | 4 | 2 | 0 | 2 |
| Other | 3 | 0 | 3 | 0 |
| Participant engaged with incorrect intervention | 5 | 2 | 3 | 0 |
| Unknown | 45 | 9 | 10 | 26 |
