# Supplementary material for: Feasibility and Preliminary Efficacy of Web-Based and Mobile Interventions for Common Mental Health Problems in Working Adults: Multi-Arm Randomized Pilot Trial
Source: JMIR Form Res. 2022 Mar 3;6(3):e34032. doi: 10.2196/34032 (PMC8931651; doi:10.2196/34032)
Supplement: Multimedia Appendix 7 [file formative_v6i3e34032_app7.docx]

# **Multimedia Appendix 7**

Sample size and baseline mean scores for subgroup analyses on secondary outcome measures. *CS* = Combatting Stress; *WW* = Working With Worry; *BR* = Building Resilience.

| Outcome | n | mean (*t0*) | SE |
| --- | --- | --- | --- |
| **PSS** |  |  |  |
| Control | 56 | 21.9 | 0.57 |
| *CS* | 59 | 21.0 | 0.52 |
| *WW* | 59 | 22.7 | 0.59 |
| *BR* | 62 | 22.3 | 0.64 |
| **GAD-7** |  |  |  |
| Control | 56 | 9.46 | 0.53 |
| *CS* | 55 | 9.51 | 0.49 |
| *WW* | 56 | 9.98 | 0.56 |
| *BR* | 60 | 10.2 | 0.58 |
| **PHQ-8** |  |  |  |
| Control | 55 | 9.85 | 0.53 |
| *CS* | 55 | 10.0 | 0.50 |
| *WW* | 59 | 10.0 | 0.58 |
| *BR* | 60 | 10.7 | 0.59 |
| **BRS** |  |  |  |
| Control | 32 | 14.1 | 0.43 |
| *CS* | 32 | 14.3 | 0.34 |
| *WW* | 43 | 14.1 | 0.33 |
| *BR* | 35 | 13.4 | 0.45 |
